# A quasi-experimental evaluation of compliant flooring in a residential care setting

**DOI:** 10.1371/journal.pone.0201290

**Published:** 2018-07-26

**Authors:** Johanna Gustavsson, Carl Bonander, Finn Nilson

**Affiliations:** Centre for Public Safety, Faculty of Health, Science and Technology, Karlstad University, Karlstad, Sweden; Berner Fachhochschule, SWITZERLAND

## Abstract

**Background:**

Fall injuries affect the lives of older people to a substantial degree. This quasi-experimental observational study investigates the potential fall injury reducing effect of a compliant flooring in a residential care setting.

**Methods:**

The allocation of the compliant flooring was non-random. Data on fall-events and individual characteristics were collected in a residential care unit during a period of 68 months. The primary outcome was the fall injury rate per fall, and a logistic regression analysis was used to test for the effect of complaint flooring. Falls per 1000 bed days was the secondary outcome, used to measure the difference in fall risk on compliant flooring versus regular flooring.

**Results:**

The event dataset is an unbalanced panel with repeated observations on 114 individuals, with 70% women. The mean age was 84.9 years of age, the average Body Mass Index (BMI) was 24.7, and there was a mean of 6.57 (SD: 15.28) falls per individual. The unadjusted effect estimate showed a non-significant relative risk injury reduction of 29% per fall (RR 0.71 [95% CI: 0.46–1.09]) compared to regular flooring. Re-estimating, excluding identified outliers, showed an injury risk reduction of 63% (RR 0.37 [95% CI: 0.25–0.54]). Falls per 1000 bed days showed that individuals living in apartments with compliant flooring had a fall rate of 5.3 per 1000 bed days compared to a fall rate of 8.4 per 1000 bed days among individuals living in regular apartments. This corresponds to an incidence rate ratio (IRR) of 0.63 (95% exact Poisson CI: 0.50–0.80).

**Conclusion:**

The results of this non-randomized study indicate that compliant flooring has the potential to reduce the risk of fall injury without increasing the fall risk among older people in a Swedish residential care setting.

## Background

Fall injuries are a substantial public health problem in the elderly population [1], with a hip fracture incidence rate of 850/100,000 for people over 65 years of age in Sweden [2]. For nursing home residents, the injury rates are considerably higher compared to the community-dwelling older people, approximately 7 times higher for women and 11 times higher for men [3]. In the last decades, substantial efforts have been made to prevent falls and fall injuries amongst older people, both within the clinical field and in health research [4–6]. However, for older patients living in residential care facilities, evidence-based interventions are still scarce [6–9]. Due to aspects such as comorbidities or frailty, programs that are effective in the community-dwelling older population may not necessarily be suitable for older people in residential care [10].

A recently published scoping review [11] found that compliant flooring, floor covering with shock absorbing capacity, may reduce fall injuries for older people in residential care, therefore supporting previous laboratorial findings [12–15]. However, Lachance et al (2017) highlight the need for further clinical research. In our study, published in 2015, we showed a 59% injury risk reduction for falls on compliant flooring compared to falls on regular flooring [16]. However, these were preliminary results with limited data merely on women. In addition to the need for further studies on the effect of compliant flooring on fall injury risk, other clinical studies have highlighted the potentially increased risk of falls with compliant flooring given the softer surface [17, 18]. The aim of this study, therefore, is to evaluate the potential fall injury reducing effect of a compliant flooring in a residential care facility for older people whilst also analysing the potentially increased risk of falling.

## Method

### Design

This study is a quasi-experimental trial evaluating the effect of compliant flooring in a natural setting, in which the municipality initiated the installation of the flooring without randomization. Given that the municipality independently installed the flooring, we had no control over the sample size in terms of how many units the floor was installed in, nor could we determine how many individuals would be included in the study.

### Setting and participants

The studied residential care unit contains 60 apartments divided into 6 identical wards, accommodating approximately 60 individuals (of which approximately 70% are women). As is common in Swedish geriatric residential care, a majority of the apartments are designed for one person, although in some situations couples can live together. Due to Swedish elderly care being regulated by the Social Services Act (SOL 2001), the availability of heavily subsidized home care, and private residential care being exceedingly rare. The population (older people living in residential care) generally have substantial cognitive and/or physical impairments, requiring considerable supervision and functional support and care [19], as this is a requirement for placement in residential care. In the studied high-level residential care facility, the residents had 24-hour access to assistance with activities of daily living and medical care.

All residents living at the studied unit were invited to participate in the study and written consent was collected, with 94% agreeing to participate. Participants were continuously recruited, based on when they moved in, during the study period. The resident’s capacity to consent was determined by the staff, based on their clinical expertise on evaluating cognitive ability. In cases where the resident could not give consent due to cognitive impairment, the next of kin were asked to determine whether the resident was to participate. This study have been approved by the Central Ethical Review Board, Uppsala. Approval number 2011/147. Written informed consent were obtained from the participant’s prior to data collection.

### Intervention

In 2011, 350 m^2^ of compliant flooring was installed in parts of one of the wards (6 out of 10 apartments, the communal dining room and parts of the corridor). During 2014, an additional 300 m^2^ of compliant flooring was installed in parts of another ward (5 of 10 apartments, the communal dining room and the corridor). The allocation of the rooms was non-random and monitored by the staff. During the study period, following initiatives from staff or relatives, four fall prone participants were moved from rooms with regular flooring to rooms with compliant flooring. Three of these participants continued to fall after they had been moved.

Flooring in the control areas were vinyl, linoleum or ceramic tiles, all with concrete underlay. Control areas were present on all six wards. These areas consisted of bedrooms, communal areas (except in one ward with compliant flooring in all communal areas), and living rooms, in similarity to the intervention areas. The compliant flooring was not installed in bathrooms as the flooring is not approved for bathroom installation.

**Fig 1:**
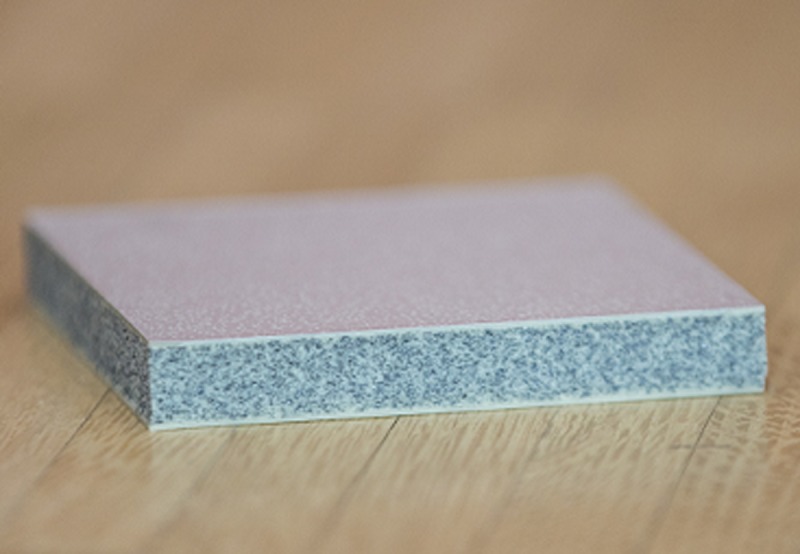
A section of the studied compliant flooring.

The installed compliant flooring, marketed under the trademark Kradal, is a 12 mm thick closed cell, flexible polyurethane/polyurea composite tile (500 x 500 mm) with an exterior surface of polyurethane/polyurea elastomers approximately 1.5 mm thick (Fig 1). The interior is closed cell flexible polyurethane. The tiles were laid onto the sub-floor (concrete) with approximately 4mm flexible grouts between tiles and then coated in order to seal the floor’s top surface.

The Kradal flooring has previously been tested in a laboratorial setting for its effect on balance, showing no difference in gait stability compared to vinyl and carpet with commercial underlay [20]. In another experiment, human volunteers were used to test for impact attenuation during lateral falls on the pelvis and whether BMI and gender modified the impact attenuation. The participant was positioned on the side with their pelvis supported by a sling 5 cm above the flooring sample, and released on to different flooring samples. The results showed an overall protective effect, especially for those with low BMI [21].

### Outcomes

#### Primary

The primary outcome was the fall injury rate per fall, which was measured using a binary indicator for whether an injury occurred during a specific fall event. The reason behind this choice of outcome was that the treatment variable could vary within individuals, between different fall events. Hence, it was the event that was assigned to treatment depending on where the individual fell, rather than the individuals themselves.

#### Secondary

Falls per 1000 bed days was used to measure the difference in fall risk for individuals living in apartments with compliant flooring compared to those living in apartments with regular flooring.

### Data collection

During the data collection, all falls were registered in accordance with the existing injury surveillance system at the residential care home and all falls were recorded on a fall registration form. A fall was defined as “an unexpected event in which the participants come to rest on the ground, floor or lower level” [22]. Within the surveillance system, information concerning the fall was collected, for example, date, location, type of flooring, activity and whether a hip protector was worn. In order to obtain more detailed information on resulting injuries, the type of injury, diagnostic tools and treatment, an injury-registration form was added in addition to the regular surveillance system. The outcomes of the fall-events were categorized as recommended [23]; ‘no injury’, ‘minor’ (minor bruises or abrasions not requiring assistance from health professional; reduction in physical function (e.g. due to pain, fear of falling) for at least three days), ‘moderate’ (wounds, bruises, sprains, cuts requiring a medical/health professional examination such as physical examination, x-ray, suture) or ‘severe’ (medically recorded fracture, head or internal injury requiring accident and emergency or inpatient treatment) with the addition of the category ‘death’. When it was clear in the fall- or injury-registration form that the injury occurred in contact with something other than the flooring, the event was excluded. Relevant individual information on age, sex, body height and weight was collected from the patient medical records by the staff. The registered individual-level covariates were: medications (sedatives/tranquillizers/neuroleptics, anti-depressives), sensory deficits, cognitive impairment (cognitive impairment or no cognitive impairment) and walking ability (safe-walker (with or without walking aid), unsafe-walker or non-walker). Event-specific covariates were: hip protectors, time during the day (6-10AM, 10AM-2PM, 2-6PM, 6-10PM, 10PM-02AM and 2-6AM), activity (low transfer (e. g. from bed to wheelchair), sitting or lying down, standing or walking and unknown) and location (bathroom, bedroom, communal areas).

Ethical approval was received from the regional ethical board at the University of Uppsala, (Registration Number 2011/147).

### Statistical analysis

When testing for the effects of complaint flooring on injury risk per fall, we used logistic regression analysis to account for the potential confounders, including age, sex, BMI, visual impairment and cognitive impairment. We also included walking ability, hip protectors, location of falls, activity when falling, and time during the day. These variables were chosen due to their potential effects on the impact force during a fall, or because they may contribute to variations in fall type [24, 25]. As usual, the results from the logistic regression models are presented as odds ratios. For increased interpretability [26], we also present risk ratios and risk differences, which were calculated using the estimated marginal means from the model [27]. Prior to the primary analysis, we also explored the differences between falls on the two flooring types with respect to these covariates, using linear regression for continuous variables and logistic regression for binary variables. A cluster-robust covariance matrix was used in all event-level analyses to adjust inferential statistics for within-individual correlation [28].

Due to the potentially strong influence of frequent fallers on the results, a sensitivity analysis was performed in which the relative change in the treatment effect estimate was measured repeatedly while iteratively leaving out one individual at a time. Individuals who influenced the treatment effect by more than Q(25)-IQR*3 in the negative direction or Q(75)+IQR*3 in the positive, where Q() is a quantile of the leave-one-out treatment effects and IQR is the interquartile range, were considered to be extreme outliers following Tukey’s definition [29]. For reference, these observations would correspond to 0.002% of a normally distributed population. The main analyses are presented both with and without these outliers in the results section.

Individual fixed effects (FE) regression model were also computed using a case-crossover sample containing n = 13 individuals who had fallen on both types of flooring using a conditional logistic regression model. Because the FE model compares individuals to themselves when they fall on different (treatment and control) floors, it effectively adjusts for unobserved individual effects that do not vary between events.

Differences in fall rates between intervention and control rooms were computed using Poisson regression models with the number of bed-days as an offset term, adjusting for age, sex, cognitive ability, walking ability, medications (antidepressants and sedatives) and visual impairment.

The analyses were performed in Stata version 12 (StataCorp, 2011).

## Results

### Descriptive statistics

**Fig 2:**
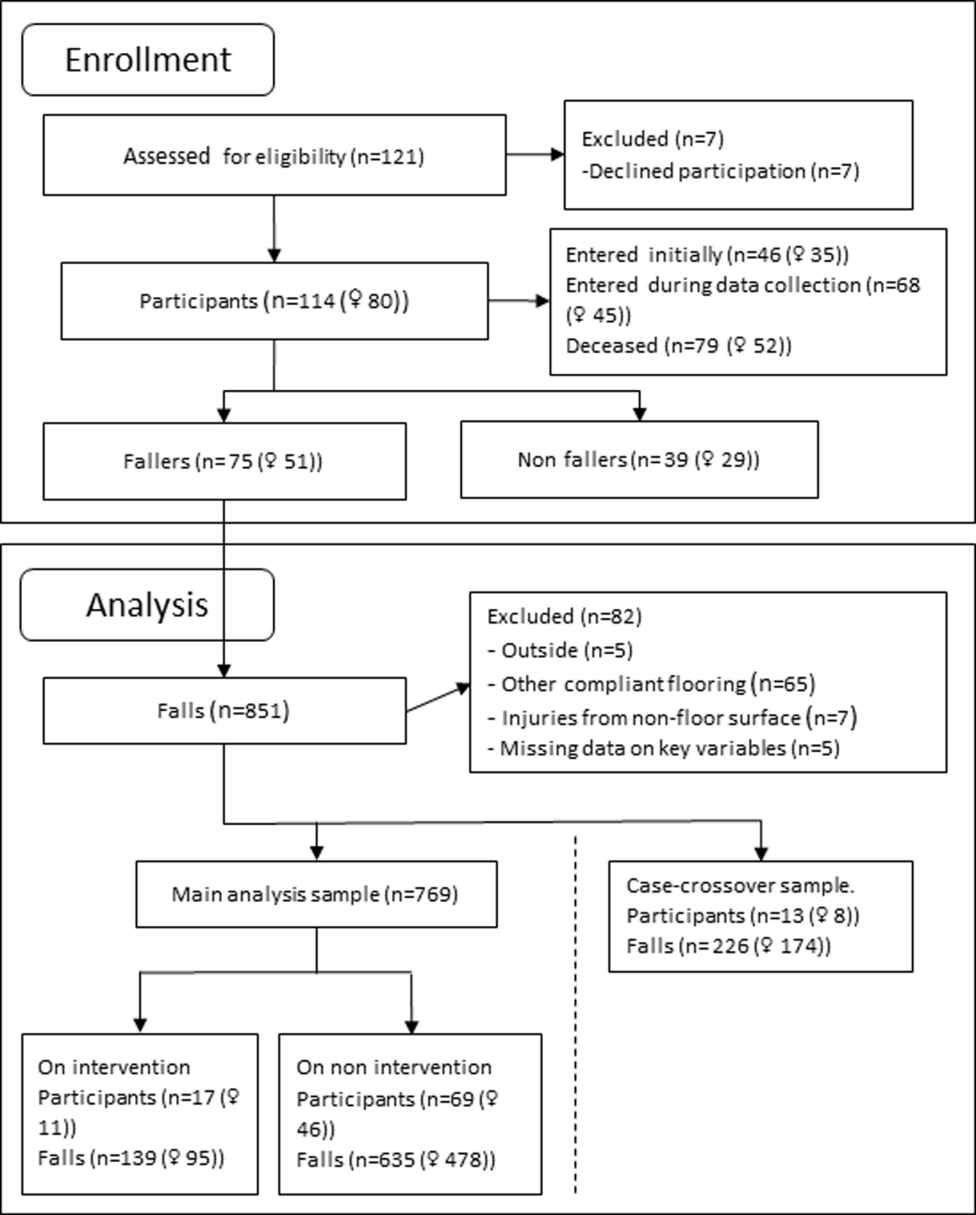
Recruitment and participant flow.

Data collection took place between October 1^st^ 2011 and May 31^st^ 2017. The event dataset is an unbalanced panel with repeated observations on n = 114 (Fig 2) individuals with a mean of 6.57 (SD: 15.28) falls per individual (Table 1). The mean age was 84.9 years of age. The average Body Mass Index (BMI) was 24.7. No participants moved out although 79 participants died and new residents moved in. Therefore, the length of stay varied from 20 to 2,069 days (Mean: 834, SD: 618) during the 68 months of data collection. The number of bed days included in the study was 95,036. Because the main outcome measured was injury risk per fall, only individuals who fell at least once were included in the injury outcome analysis. Of all the participants included in the study, 66% fell at least once. A comparison between the entire sample and the analysis sample is available in Table 1.

**Table 1:** Descriptive statistics for all participants and fallers with at least one fall.

	All	Fallers
Participants (% of total)	114	75 (66)
Age (m (SD))	84.88 (6.5)	85.29 (6.3)
BMI (kg/m^2^) (m (SD))	24.7 (4.7)	25.7 (4.8)
Women (n (%))	80 (70.2)	51 (68.0)
Visual impairment (n (%))	53 (46.5) (4 missing)	34 (45.9)(1 missing)
Cognitive impairment (n (%))	57 (50) (4 missing)	45 (60.8) (1 missing)
Sedatives/Tranquillizers/Neuroleptics (n (%))	50 (43.9) (5 missing)	38 (51.3) (1 missing)
Anti-depressive (n (%))	49 (43) (4 missing)	31 (41.3)
Walking ability (n (%))		
*Safe-walker*	33 (28.9) (6 missing)	23 (31.1) (1 missing)
*Unsafe-walker*	51 (44.7)	42 (56.8)
*Non-walker*	24 (21.1)	9 (12.2)
Downton estimates (m (SD))	4.41 (1.64)	4.73 (1.41)
Total number of falls	851	-
Falls with hip protectors (n (%))	114 (13.4)	-
Falls/1000 bed days	9.0	-
Bed days (n, range, m (SD))	95036, 20–2069, 834 (618)	-

Abbreviation descriptions: m = mean, SD = standard deviation.

In total, 851 falls were recorded and the fall rate was 9 per 1,000 bed days (Table 1). Sixty-five falls occurred on other surfaces than the two compared in this study (mattress and fall mat), five falls occurred outside and 7 falls were excluded as the injury outcome was caused by something other than flooring, leaving 774 fall events relevant to include in the analysis.

Falls occurred both in the residents apartments and in communal areas. The falls mainly occured in bedrooms (72%), followed by bathrooms (15%), and communal areas (13%). The most common activity before falling was walking or transfering (e.g. from bed to chair). A majority of the fall events occurred on regular flooring. The crude injury risk per fall was 28.7% (31.9% for women and 19.1% for men) for falls on regular flooring, and 20.3% (21.1% for women and 18.6% for men) for falls on compliant flooring (Table 2). Most injuries were minor (82%), and too few moderate and severe injuries occurred to analyze differences in effectiveness by severity (Table 2).

**Table 2:** Injury severity distribution by type of flooring.

	Falls on compliant flooring	Falls on regular flooring
All falls (n)	138	631
No injury (n (%))	110 (79.7)	450 (71.3)
All falls with injuries (n (%))	28 (20.3)	181 (28.7)
Minor (n (%))	23 (16.7)	148 (23.5)
Moderate (n (%))	2 (1.4)	18 (2.9)
Major (n (%))	3 (2.2)	15 (2.4)
Death (n (%))	0	0

Table 3 displays a covariate balance check between falls on compliant and regular flooring. The only statistically significant difference found was that falls on regular flooring had a larger proportion of unknown activity reports. Another difference, that we could not formally test using logistic regression, is that no falls on compliant flooring occurred in bathrooms (because Kradal was not installed in bathrooms).

**Table 3:** A covariate balance check for falls on compliant versus regular flooring.

Variable (measure, m = mean, p = proportion)	Falls on compliant flooring	Falls on regular flooring	Difference (compliant-regular)	P-value for difference
Age (m)	85.0	82.8	2.17	0.225
Women (p)	0.68	0.75	-0.07	0.686
BMI (m)	23.8	24.4	-0.60	0.526
Visual impairment (p)	0.29	0.31	-0.02	0.930
Cognitive impairment (p)	0.73	0.67	0.06	0.773
Hip protector (p)	0.18	0.14	0.04	0.750
Walking ability (p)				
*Safe walker*	0.34	0.49	-0.15	0.548
*Unsafe walker*	0.63	0.45	0.18	0.445
*Non-walker*	0.04	0.07	-0.03	0.498
Location (p)				
*Bathroom*	0.00	0.19	-0.19	N/A^a^
*Bedroom*	0.81	0.69	0.12	0.328
*Communal area*	0.19	0.12	0.07	0.431
Activity (p)				
*Low transfer (e*.*g*. *bed to wheelchair)*	0.41	0.29	0.12	0.064
*Sitting or lying down*	0.13	0.10	0.03	0.374
*Standing or walking*	0.33	0.39	-0.06	0.448
*Unknown*	0.13	0.22	-0.09	0.002
Time of day (p)				
*6-10AM*	0.13	0.12	0.01	0.810
*10AM-2PM*	0.13	0.15	-0.02	0.610
*2-6PM*	0.19	0.22	-0.02	0.632
*6-10PM*	0.17	0.21	-0.03	0.481
*10PM-02AM*	0.20	0.16	0.04	0.547
*2-6AM*	0.17	0.14	0.03	0.427


Models were fitted separately for each variable. Categorical variables were transformed to dummy variables for each category prior to estimation. Continuous variables were analyzed using linear regression, and binary variables (presented as proportions) were analyzed using logistic regression (differences from these models are presented as marginal means obtained using the *margins* command in Stata 12). All models were fitted using clustered standard errors to account for within-individual correlation.

^a^A logistic model could not be fitted to the data, because the Kradal flooring was not installed in bathrooms.

### Primary results

The unadjusted estimate of the effect of compliant flooring showed a non-significant relative risk reduction of 29% per fall compared to regular flooring, which corresponds to a risk difference of -0.084 percentage points. Adjusting for potential confounders did not affect the results to any meaningful extent (Table 4), nor did excluding bathrooms from the control floors (adjusted risk ratio: 0.73, 95% CI: 0.49, 1.08; adjusted risk difference: -0.08, 95% CI: -0.183, 0.020).

**Table 4:** Logistic regression results for the effect of complaint flooring on injury risk per fall.

	Unadjusted estimates (95% CI)	Adjusted estimates (95% CI)^a^
Odds ratio (OR)	0.63 (0.36, 1.12)	0.62 (0.36, 1.07)
p-value	0.12	0.085
Risk on complaint flooring	0.20 (0.12, 0.29)	0.21 (0.12, 0.29)
Risk on regular flooring	0.29 (0.24, 0.34)	0.29 (0.25, 0.33)
Risk ratio (RR)	0.71 (0.46, 1.09)	0.71 (0.48, 1.05)
Risk difference (RD)	-0.084 (-0.189, 0.022)	-0.085 (-0.181, 0.012)


^*a*^The covariates included in the adjusted analysis were age, sex, BMI, visual impairment, cognitive impairment, walking ability, hip protectors, location of the fall (room type), activity when falling, and time of day.

#### Sensitivity analysis

**Fig 3:**
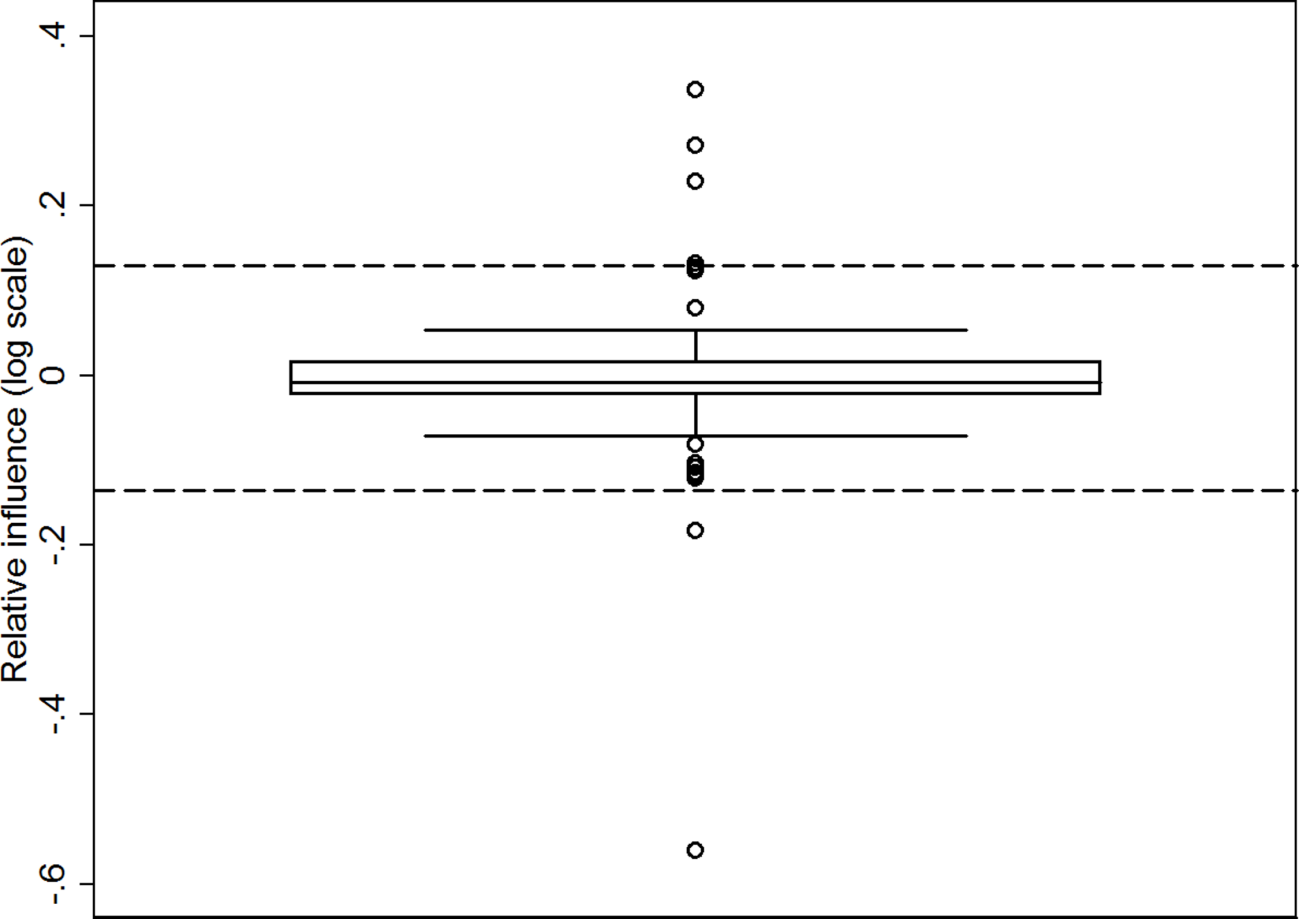
Box plot of the relative influence of each individual on the treatment effect estimate. Extreme outliers are shown as the dots below or above the dashed lines.

The outlier detection analysis found two individuals who, once removed, influenced the effect estimate strongly in the negative direction, and four who influenced the results in the positive direction (Fig 3). Re-estimating the analyses presented in Table 4 without these individuals yields a very different result. The unadjusted estimate without the outliers showed an injury risk reduction of 63% (RR 0.37 [95% CI: 0.25–0.54]). As in Table 4, the adjusted estimate was almost identical (RR 0.39 [95% CI: 0.24–0.63]).

### Secondary analyses

#### Sex-stratified results

The crude injury rate indicated that women were at higher risk of injury when falling on regular flooring compared to men, which suggests that the baseline risk is lower for men. For men, the injury rate was similar on both flooring types (regular flooring: 19.1%, compliant flooring: 18.6%), which in both instances is comparable to the rate for women on compliant flooring. The unadjusted RR was 0.66 (95% CI: 0.39–1.13) for women and 0.97 (95% CI: 0.51–1.87) for men. These numbers correspond to risk differences (RD) of -0.108 (95% CI: -0.248, 0.032) and -0.005 (95% CI: -0.128, 0.118) for women and men, respectively. The result for women was very similar even after adjustment for the same covariates used in the main analysis (RR: 0.67 [95% CI: 0.43–1.03], RD: -0.017 [95% CI: -0.222, 0.008]). For men, the point estimates were further from the null than in the unadjusted analysis (RR: 0.73 [95% CI: 0.40–1.33], RD: -0.055 [95% CI: -0.160, 0.050]).

As in the main analysis, the results change considerably when excluding the extreme outliers (RR 0.35 [95% CI: 0.17–0.73] for women; RR 0.24 [95% CI: 0.06–0.85] for men).

#### Case-crossover sample

Thirteen participants fell on both surfaces and could be used in the case-crossover analysis. Together, this subsample fell 226 times during the study. The result from an individual fixed-effects logistic regression model on this data showed a non-significant risk reduction of 38% (RR 0.62 [95% CI: 0.36–1.08]), and a risk difference of -0.125 (95% CI: -0.270, 0.019) between the treatment and control floors.

#### Differences in fall rates between intervention and control rooms

Dividing the sample into two groups based on apartment type (compliant or regular flooring) and comparing their fall rates per 1,000 bed days showed that individuals living in apartments with compliant flooring had a fall rate of 5.3 per 1,000 bed days compared to a fall rate of 8.4 per 1,000 bed days among individuals living in regular apartments. This corresponds to an incidence rate ratio (IRR) of 0.63 (95% exact Poisson CI: 0.50–0.80). The results did not change significantly after adjustment for age, sex, cognitive ability, walking ability, medications (antidepressants and sedatives) and visual impairment in a Poisson regression model (IRR 0.64 [95% CI: 0.51–0.82]). Because the exposure data (bed-days) cannot be separated into time spent within and outside the bedroom, it is impossible to be certain that these numbers do not reflect different exposure patterns. Hence, we also performed analyses excluding all events apart from those that occurred in the bedrooms, for which it can be inferred that the individual walked on either the intervention or control flooring at the time of the fall. This did not affect the results (adjusted IRR 0.56 [95% CI: 0.40–0.77]).

## Discussion

The aim of this study was to investigate the potential fall injury reducing effect of compliant flooring in a residential care population, as well as study the effect on fall risk. The results indicate that the compliant flooring has the potential to reduce the risk of fall injury, and there were no signs of an increased fall risk in our sample.

However, the results are in some aspects inconclusive and there is a larger variance in the current data compared to an interim analysis of the same compliant flooring and setting [16]. After collecting data for more than twice as long, we arrived at similar point estimates, but with lower precision. We assume this is caused by the data structure, which allowed for multiple events per individual. In this case, this aspect posed a challenge in terms of power and sample size calculations.

The primary model showed a non-significant 29% injury risk reduction for falls on compliant flooring compared to falls on regular flooring. However, when excluding the six outliers, the results showed a significant reduction with 63%, which is comparable to our interim analysis of the data (59%) [16], and in line with previous results showing a 42% non-significant decrease in injury rate [17]. Also, the case-crossover results also showed a tendency towards an injury-reducing effect, although the point estimates were smaller. The results from the primary analysis and the sensitivity analysis result in different conclusions regarding the efficacy of the intervention, which motivates some clarification and discussion about how the two estimates can be interpreted. Assuming the sample of individuals used in the primary analysis represent that of the average residential care facility in Sweden, it could be argued that a few highly influential individuals (outliers), in terms of the overall impact of the intervention, are to be expected. If this is the case, then the primary result is the most policy relevant estimate of the intervention effect. If, instead, the outliers are unique to the studied residential care facility, or if we observe them as outliers due to errors in data collection, then it could be argued that the result from the sensitivity analysis is the most policy relevant estimate. Even if the latter points are untrue and the sample does reflect that of the average residential care facility, the estimate from the sensitivity analysis could be interpreted as an estimator for the expected average effect on the majority of residents in Swedish residential care facilities. Hence, it may still make sense, from an individual perspective, to consider the estimate from the sensitivity analysis. Unfortunately, our data do not permit us to fully answer why these individuals change the results so drastically, but it could be hypothesized that it is the product of unobserved effect modifiers. It could therefore be useful for future studies to examine heterogeneous effects to identify for whom the intervention works, as this would allow for informed individualized treatment decisions.

In our previous study, we could only credibly estimate effects for women as there were too few men in the study at the time. The unadjusted sex-stratified results presented in this paper show that the injury risk per fall for men is at approximately the same level independent of flooring type. Notably, their injury risk on regular flooring is comparable to that of the women who fell on compliant flooring, suggesting that the baseline risk is lower for men. The idea that men have a lower injury risk rate per fall is supported by previous research [24, 30, 31] and this may result in a ceiling effect, therefore explaining why there were no indications of an effect for this subgroup in the main result. Still, when we excluded the outliers, the results showed significant risk reductions for both men and women. To our knowledge, no other clinical studies have investigated if the effects of compliant flooring are moderated by sex, but the results from a laboratorial study showed that the biomechanical effectiveness of compliant floors was greater for men compared with women [21]. This is in conflict with our main results, but not with the results from the sex-stratified sensitivity analysis, and may need to be explored further in future studies.

In a pilot study, Drahota et al (2013) reported a non-significant increased risk of falls on Tarkett Excell Omnisport flooring in a hospital setting. Other studies have not reported this aspect [32] or have not reported any significant differences in fall risk between compliant and regular flooring types [33]. Whilst our results should be interpreted with some caution, since the exact exposure time per flooring type could not be measured, our results show a lower fall rate per 1,000 bed days for residents living in rooms with compliant flooring. The effects of complaint flooring on gait and balance have also been tested in laboratorial settings, and there are no signs that it would have an adverse effect on fall risk [13, 20].

Together with other recent publications [11, 33, 34], the results from this study add to the growing body of evidence that supports compliant flooring as an effective approach to reducing the risk of fall injuries among older people. It is an intervention that has the benefit of being a passive measure that circumvents individual barriers to compliance, and even if it does not eliminate the risk of fall injuries entirely, it has the potential to be a relevant contribution to fall injury prevention for frail older people in residential care. Whilst largely positive, there are still some barriers to implementation that need to be addressed, for example the issue of compliant flooring increasing the push and pull resistance for staff and residents [11, 35]. Adding to the complexity, there is also increased costs to installing compliant flooring. When modeling the cost-effectiveness of the studied flooring the results indicate that it can reduce costs and increase QALYs. However, this has to be viewed in a societal perspective as one societal actor bears the cost of instalment (municipalities), whilst another benefits from the effects of reduced hip fracture costs (county councils) [34]. This could potentially pose as a barrier to wide-spread adoption of the intervention, and is something that needs to be considered in future studies.

### Strengths and limitations

There are inherent difficulties with experimental trials within injury research due to aspects related to both ethics and costs [36]. It is therefore important to use the possibility that lie in evaluating implementations of interventions initiated by societal actors in order to produce new evidence. However, there are some limitations to this approach worth mentioning. The largest threat to the internal validity of this study is that the intervention was not randomized. There is a risk of bias, as the staff was responsible of the allocation of residents. During the data collection, four residents were moved to compliant flooring due to a perceived increased fall risk (personal communication). However, the remainder of the individuals who entered the study after the flooring was installed were assigned to rooms based on availability, which in itself may produce some pseudo-randomization. As the study was non-blinded there is also a risk of ascertainment bias. The staff that reported the outcome of the fall had knowledge of the fall surface and given that 82% of the injuries were minor, with the only diagnostic tool being subjective observation, there are some risks. In general, non-blinded outcome assessment can result in an overestimation of treatment effects [37], which may also be the case here. Unfortunately, it was practically infeasible to blind the staff to treatment assignment.

This notion is supported by the fact that the effect estimates did not change when covariates were included, and that the case-crossover sample (which adjusts for unobservable individual effects) showed similar results.

Another limitation is that we were unable to present results showing differences in injury severity divided by flooring due to the study being underpowered for such detailed analyses. This is problematic as we cannot distinguish between effects on bruising and fractures, the latter being the more clinically relevant outcome. For this, a more large-scale study will be needed, such as the experiment currently conducted by Robinovitch and colleague [38]. Our data collection has now ended, but the data from this study could be pooled with data from other trials to gain additional power and precision.

## Conclusions

Complaint flooring seems to reduce the risk of fall injuries in residential care for the older people and our results do not indicate that the studied flooring increases the risk of falling. There is a need for further research comparing and evaluating different types of flooring solutions in order to reach a more optimal impact-absorbing effect whilst simultaneously balancing other functions of a flooring in a care environment.
